# Overview of Scorpion Species from China and Their Toxins

**DOI:** 10.3390/toxins6030796

**Published:** 2014-02-26

**Authors:** Zhijian Cao, Zhiyong Di, Yingliang Wu, Wenxin Li

**Affiliations:** State Key Laboratory of Virology, College of Life Sciences, Wuhan University, Wuhan 430072, China; E-Mails: zjcao@whu.edu.cn (Z.C.); scorpionking@whu.edu.cn (Z.D.)

**Keywords:** scorpion, fauna, toxin, function, evolution, relationship

## Abstract

Scorpions are one of the most ancient groups of terrestrial animals. They have maintained a steady morphology over more than 400 million years of evolution. Their venom arsenals for capturing prey and defending against predators may play a critical role in their ancient and conservative appearance. In the current review, we present the scorpion fauna of China: 53 species covering five families and 12 genera. We also systematically list toxins or genes from Chinese scorpion species, involving eight species covering four families. Furthermore, we review the diverse functions of typical toxins from Chinese scorpion species, involving Na^+^ channel modulators, K^+^ channel blockers, antimicrobial peptides and protease inhibitors. Using scorpion species and their toxins from China as an example, we build the bridge between scorpion species and their toxins, which helps us to understand the molecular and functional diversity of scorpion venom arsenal, the dynamic and functional evolution of scorpion toxins, and the potential relationships of scorpion species and their toxins.

Scorpions are one of the most ancient groups of terrestrial animals, belonging to the class Arachnida within the phylum Arthropoda. Scorpions represent a basal branch of arachnids and have a relatively distant relationship with Acari (mites) and Araneae (spiders), the other two groups of the class Arachnida. Thus, scorpions have an important phylogenetic position within the phylum Arthropoda and the class Arachnida (Figure 1) [1]. There are approximately 15 families, 197 genera and 2,089 species recorded in the world except for Greenland and Antarctica (http://www.ntnu.no/ub/scorpion-files/index.php, accessed on 8 October 2013).

**Figure 1:**
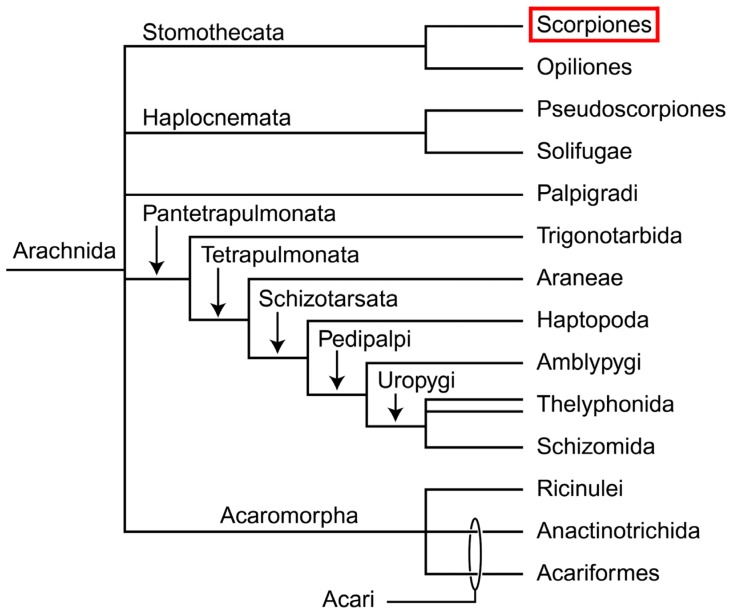
Phylogenetic position of the scorpiones within the class Arachnida [1].

Scorpions are notorious for their deadly venoms. On the one hand, scorpion envenomation is a significant threat to public health in many regions around the world, which is a major cause of mortality in some underdeveloped countries in Latin America, South America, the Indian Subcontinent, the Middle East, and Africa. The number of worldwide scorpion stings is estimated to be around 1.2 million annually, resulting in more than 3,200 deaths [2]. On the other hand, scorpions developed their venom system as a primary weapon for capturing prey and defending against predators. Their venom systems contain the large families of toxins with a broad biodiversity [3].

Studies on the systematical classification of scorpion species from around the world and from specific countries have recently been garnering a great deal of attention [4,5,6,7,8]. Scorpion toxins have also attracted the fervent attention of a considerable number of researchers, due to the potential for developing therapeutic drugs [9,10,11]. In this review, we present the scorpion fauna of China. Additionally, scorpion toxins or genes from China are listed systematically. Using scorpion species and their toxins from China as an example, we try to build the bridge between scorpion species and their toxins, which helps us to not only understand the relationship of scorpion species and their toxins, but also show insights into the dynamic and functional evolution of the scorpion venom arsenal.

## 1. Scorpion Species from China

Chinese scorpion taxonomy research was started by the visiting scientists, dating back to 1879 with the first description of a Chinese scorpion (*Buthus martensii* Karsch also means *Mesobuthus* *martensii* Karsch, see the review [12]). There were 19 species and subspecies reported before 2003 [12], covering five families and nine genera. Since then, some Chinese researchers began to study scorpion classification and identification in China, particularly two representative groups: Minsheng Zhu team from Hebei University and our group. Qi *et al*. published the first comprehensive report on scorpions from Xizang, and discovered eight new species belonging to the families Chaerilidae (*Chaerilus*) and Euscorpiidae (*Euscorpiops* and *Scorpiops*) [13]. Subsequently, new genera, new species, a new record of genera and species, and re-descriptions were reported continuously [14,15,16,17,18,19,20,21,22,23,24,25,26,27,28,29,30,31,32,33,34,35,36,37]. At the same time, some revisions on scorpion distribution and taxonomy of the genera were completed [16,20,29,30,33,34,35,36]. A recent report began to focus on the impact of climate and environmental changes on the evolution and formation of scorpion species from China [38].

Currently, 53 species of 12 genera of five families (Buthidae, Chaerilidae, Euscorpiidae, Hemiscorpiidae and Scorpionidae) are recorded in China: (Table 1). Buthidae (Buthidae C. L. Koch, 1837) is the most widely distributed family in China, including six genera (*Hottentotta* with two species, *Isometrus* with three species, *Lychas* with two species, *Mesobuthus* with nine species, *Orthochirus* with one species, and *Razianus* with one species) and 18 species and subspecies (Table 1). They are distributed across most provinces except for Guangdong, Guizhou, Heilongjiang, Hunan, Jiangsu, Jilin, and Sichuan. Chaerilidae (Chaerilidae Pocock, 1893) has just a monotype genus Chaerilus, and all eight of the chaerilid species are found in South Xizang. Euscorpiidae (Euscorpiidae Laurie, 1896) is the largest family in China and has two genera *Euscorpiops* (11) and *Scorpiops* (11) mainly found in Yunnan and Xizang. Di *et al*. reported the *Scorpiops* species distributed in Central China [36], which is the first record of the family Euscorpiidae (Arachnida: Scorpiones) from Central China [36]. Hemiscorpiidae (Hemiscorpiidae Pocock, 1893) has two genera (*Liocheles* with one species and *Tibetiomachus* with one species) from China. *Liocheles* species is found on Hainan island. Lourenço and Qi described one new genus (*Tibetiomachus* Lourenço and Qi, 2006 (Hemiscorpiidae)) and new species (*Tibetiomachus himalayensis* Lourenço and Qi, 2006) based on specimens from Xizang [23]. *T*. *himalayensis* is the first liochelid scorpion found in the high Himalayan Mountains of Xizang, and also the first example of the family to be collected out of its typical tropical and subtropical areas of distribution [23]. Scorpionidae (Scorpionidae Latreille, 1802) has one genus (*Heterometrus*) and three species reported in China: *H*. *tibetanus* found in Xizang but the other two species (*H*. *longimanus* and *H*. *petersii*) without an exact distribution area.

**Table 1:** Catalog of scorpion species from China.

Family	Genera	Species (endemic)	Distribution	References
Buthidae	*Hottentotta*	*Hottentotta alticola* (Pocock, 1895)	▲ 1	[12]
		*Hottentotta songi* (Lourenço, Qi and Zhu, 2005)	Xizang (endemic)	[17,24]
	*Isometrus*	*Isometrus maculatus* (DeGeer, 1778)	Hainan and Taiwan	[12,30]
		*Isometrus* *hainanensis* Lourenço, Qi and Zhu, 2005	Hainan (endemic)	[12,30]
		*Isometrus* *tibetanus* Zhu and Lourenço, 2008	Xizang (endemic)	[37]
	*Lychas*	*Lychas mucronatus* (Fabricius, 1798)	Guangxi, Hainan and Yunnan	[30]
		*Lychas scutilus* C. L. Koch, 1845	Shanghai	[12]
Buthidae	*Mesobuthus*	*Mesobuthus bolensis* Sun, Zhu and Lourenço, 2010	Xinjiang (endemic)	[17]
		*Mesobuthus caucasicus intermedius* (Birula, 1897)	Xinjiang	[12]
		*Mesobuthus caucasicus przewalskii* (Birula, 1897)	Xinjiang	[12]
		*Mesobuthus eupeus mongolcus* (Birula, 1911)	Gansu, Inner Mongolia (Neimenggu) and Ningxia	[20]
		*Mesobuthus eupeus thersites* (C. L. Koch, 1839)	Xinjiang	[20]
		*Mesobuthus karshius* Sun and Sun, 2011	Xinjiang (endemic)	[20]
		*Mesobuthus longichelus* Sun and Zhu, 2010	Xinjiang (endemic)	[19]
		*Mesobuthus martensii martensii* (Karsch, 1879)	the south side of 43°N and the north side of the Yangtze River, bordered by the Helan Mountains and the Tengger and Mo Us sand desert in the west and limited by the sea in the east	[39]
		*Mesobuthus martensii hainanensis* (Birula, 1904)	Hainan (endemic)	[12]
	*Orthochirus*	*Orthochirus scrobiculosus* (Grube, 1873)	Northwest	[40]
	*Razianus*	*Razianus xinjianganus* Lourenço, Sun and Zhu, 2010	Xinjiang (endemic)	[25]
Chaerilidae	*Chaerilus*	*Chaerilus conchiformus* Zhu, Han & Lourenço, 2008	Xizang (endemic)	[16]
		*Chaerilus dibangvalleycus* Bastawade, 2006	Xizang (endemic)	[34]
		*Chaerilus mainlingensis* Di and Zhu, 2009	Xizang (endemic)	[28]
		*Chaerilus pictus* (Pocock, 1890)	Xizang	[12]
		*Chaerilus tessellatus* Qi, Zhu and Lourenço, 2005	Xizang (endemic)	[13,34]
		*Chaerilus tricostatus* Pocock, 1899	Xizang	[34]
		*Chaerilus tryznai* Kovařík, 2000	Xizang (endemic)	[34]
		*Chaerilus wrzecionkoi* Kovařík, 2012	Xizang (endemic)	[26]
Euscorpiidae	*Euscorpiops*	*Euscorpiops asthenurus* (Pocock, 1900)	*Xizang*	[29]
		*Euscorpiops kamengensis* Bastawade, 2006	*Xizang* (endemic)	[29]
Chaerilidae	*Chaerilus*	*Euscorpiops karschi* Qi, Zhu and Lourenço, 2005	Xizang (endemic)	[29]
		*Euscorpiops kubani* Kovařík, 2004	Yunnan	[35]
		*Euscorpiops novaki* Kovařík, 2005	Xizang (endemic)	[29]
		*Euscorpiops puerensis* Di, Wu, Cao, Xiao and Li, 2010	Yunnan (endemic)	[35]
		*Euscorpiops shidian* Qi, Zhu and Lourenço, 2005	Yunnan (endemic)	[35]
		*Euscorpiops vachoni* Qi, Zhu and Lourenço, 2005	Yunnan (endemic)	[35]
		*Euscorpiops validus* Di, Cao, Wu and Li, 2010	Yunnan (endemic)	[35]
		*Euscorpiops xui* Sun and Zhu, 2010	Yunnan (endemic)	[35]
		*Euscorpiops yangi* Zhu, Zhang and Lourenço, 2007	Yunnan (endemic)	[35]
	*Scorpiops*	*Scorpiops atomatus* Qi, Zhu and Lourenço, 2005	Xizang (endemic)	[36]
		*Scorpiops hardwickii* (Gervais, 1843)	Xizang	[12,36]
		*Scorpiops jendeki* Kovařík, 1994	Yunnan (endemic)	[12,36]
		*Scorpiops langxian* Qi, Zhu and Lourenço, 2005	Xizang (endemic)	[36]
		*Scorpiops leptochirus* Pcock, 1893	Xizang	[36]
		*Scorpiops lhasa* Di and Zhu, 2009	Xizang (endemic)	[27,36]
		*Scorpiops* *luridus* Qi, Zhu and Lourenço, 2005	Xizang (endemic)	[13,36]
		*Scorpiops margerisonae* Kovařík, 2000	Xizang (endemic)	[12,36]
		*Scorpiops petersii* Pocock, 1893	Xizang	[12,33]
		*Scorpiops pococki* Qi, Zhu and Lourenço, 2005	Xizang (endemic)	[13,33]
		*Scorpiops tibetanus* Hirst, 1911	Xizang (endemic)	[12,33]
Hemiscorpiidae	*Liocheles*	*Liocheles australasiae* (Fabricius, 1775)	Hainan	[12,30]
	*Tibetiomachus* (endemic)	*Tibetiomachus himalayensis* Lourenço and Qi, 2006	Xizang (endemic)	[23]
Scorpionidae	*Heterometrus*	*Heterometrus longimanus* (Herbst, 1800)	▲ 2	[12]
		*Heterometrus tibetanus* Lourenço, Qi and Zhu, 2005	Xizang (endemic)	[24]
		*Heterometrus petersii* (Thorell, 1876)	▲ 3	[12]
5	12 (1)	53 (33)		

▲ 1–3: the localities of these species are indecisive.

## 2. Toxins from Chinese Scorpion Species

Scorpion venom has a large variety of biologically active components, most of which are the toxic peptide/protein (usually called toxins) of 1,000–9,000 Da in size [41,42]. Previous research has shown that the venom of a scorpion species has hundreds of different toxins, highlighting the large diversity of toxins in scorpion venom. Whereas most scorpion toxins have similar structural and functional characteristics, the individual components are in low abundance in the venom. Thus, it was difficult for us to isolate and purify the different single fractions from scorpion venom by the classic biochemical methods and techniques. It was therefore necessary to develop new methods or techniques for finding news toxins in scorpion venom and revealing the molecular diversity of scorpion toxins. More recently, some research groups made full use of proteomic and transcriptomic approaches to profile the venom toxin composition, which indicated that the general compositions of scorpion venoms vary among different families, genera, species and individuals [43,44,45,46]. Especially transcriptome analysis is a powerful approach not only to identify putative venom components, but also to better understand the biology of the venom gland [47,48], which was widely adopted by the research groups involving in scorpion venoms from the global world. There are several groups making use of such strategy and measure in this field, among which are the Lourival D Possani team from Mexico (National Autonomous University) and our team from China (Wuhan University) (Table 2).

**Table 2:** Summary of scorpion species with the venom transcriptomic or proteomic analysis from the world.

Family	Species	Transcriptomic analysis	Proteomic analysis	References
Buthidae	*Lychas mucronatus*	+		[48,73]
	*Isometrus maculates*			[48]
	*Buthus martensii* (=*M*. *martensii*)		+	[49]
	*Hottentotta judaicus*	+		[77]
	*Tityus discrepans*	+	+	[43,78]
	*Tityus stigmurus*	+	+	[79,80]
	*Tityus cambridgei*		+	[81]
	*Tityus costatus*		+	[82]
	*Tityus pachyurus*		+	[83]
	*Androctonus crassicauda*	+	+	[84]
	*Androctonus mauretanicus mauretanicus*		+	[85]
Scorpionidae	*Heterometrus petersii*	+	+	[45]
	*Heterometrus longimanus*		+	[86]
	*Pandinus cavimanus*	+	+	[87]
	*Urodacus yaschenkoi*	+	+	[88]
Euscorpiidae	*Scorpiops jendeki*	+		[72]
	*Scorpiops margerisonae*	+		[48]
Hemiscorpiidae	*Liocheles australasiae*	+		[89]
(Liochelidae)	*Opisthacanthus cayaporum*	+	+	[90,91]
Caraboctonidae	*Hadrurus gertschi*	+		[47]
Chaerilidae	*Chaerilus tricostatus*	+		[75]
	*Chaerilus tryznai*	+		[75]

**Figure 2:**
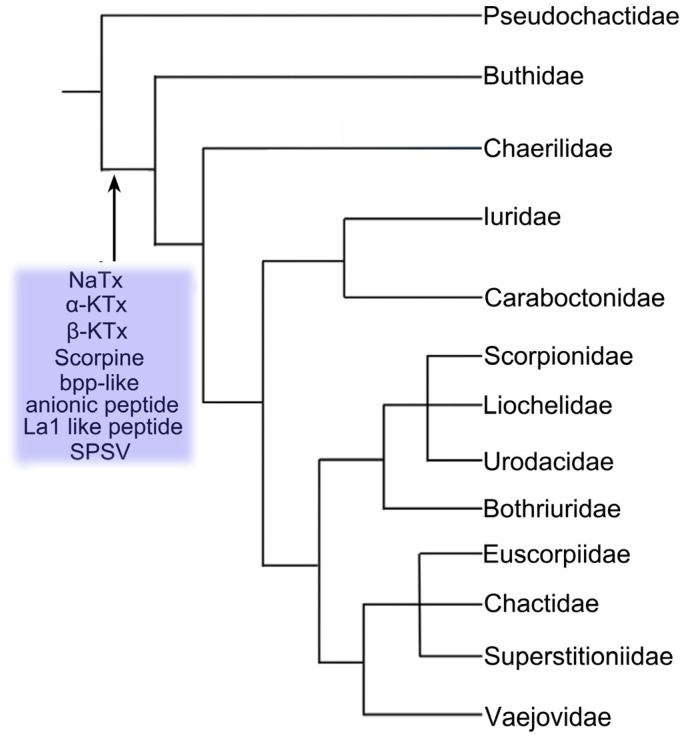
Recruitment patterns of toxin types into the scorpion venom arsenal [48,75].

In the 1990s, our lab in China started to construct the first venomous gland cDNA library of the scorpion *M*. *martensii* [49]; *M*. *martensii* has been extensively studied due to its large biomass and its usage as raw material in traditional Chinese medicine for many centuries to treat various diseases such as apoplexy, epilepsy, rheumatoid arthritis, and chronic pain [50]. From the *M*. *martensii* library, a large number of putative toxins have been described, including NaTx (toxins specific for the sodium channels) [51,52,53,54,55,56], KTx (toxins specific for the potassium channels) [57,58,59,60,61,62,63], ClTx (toxins specific for chloride channels) [64], CaTx (toxins for ryanodine receptors) [65], Bpp-like (bradykinin-potentiating peptide-like) peptide [66], cytolytic peptide [67,68], and other functional peptides [69,70,71]. In 2009, Ma *et al*. conducted a transcriptomic analysis of the venom gland of *Scorpiops jendeki* [72], which is classified to be one member of the family Euscorpiidae and whose venom has never before been investigated. This work revealed that the venom of the scorpion *S*. *jendeki* has at least 10 known toxin types (α-KTx (α subfamily of toxin specific for the potassium channel), scorpine-like peptide, calcine (toxin specific for ryanodine receptor), cytolytic peptide, TIL (trypsin-inhibitor-like) peptide, lysozyme, La1-like peptide, opistoporin-like peptide, anionic peptide, and SPSV (serine protease from scorpion venom)) and nine atypical types of peptide/protein [72]. In 2010, the toxin components from the venom of the scorpion *H*. *petersii* were evaluated by both transcriptomic and proteomic analyses [45], which resulted in the discovery of 10 known and 12 atypical types of venom peptide/protein from this scorpion venom. The 10 known types of venom peptide/protein include two types of potassium channel toxins (α-KTx and κ-KTx (κ subfamily of toxins specific for potassium channels)), four types of antimicrobial and cytolytic peptides (scorpine-like peptides, cytolytic peptide, opistoporin-like peptide and Pin2-like peptide), and one type for each of calcine, La1-like peptide, phospholipase A2, and SPSV. The 12 atypical families are composed of acid phosphatase, diuretic peptide and ten orphan families. During the same year, our group provided a comparative analysis of the venom transcriptome of the scorpion *Lychas mucronatus* from different geographical regions in China: one region is Hainan province and the other is Yunnan province [73]. Interestingly, this work identified a large number of new venom molecules, and also revealed the fact that the venom peptide/protein of the same scorpion species from different geographical regions is highly diversified. Intraspecies variation of venom composition has been more recently confirmed for the case of *Tityus serrulatus* [74]. In 2012, our lab studied the venom gland transcriptome analyses of three scorpion species: two Buthidae species (*L*. *mucronatus* and *Isometrus maculatus*) and one Euscorpiidae species (*Scorpiops margerisonae*) [48]. Transcriptomic analysis of these three scorpion species venom glands revealed 14 known types of venom peptide/protein, covering NaTx, α-KTx, β-KTx (β subfamily of toxin specific for potassium channel) and scorpine-like peptide, calcine, LVP (lipolysis-activating peptide), BPP-like (bradykinin-potentiating peptide-like), BmKb1-like peptide, cytolytic peptide, pandinin-1-like peptide, pandinin-2-like peptide, anionic peptide, La1-like peptide and SPSV. Comparative transcriptome and molecular phylogenetic analyses elucidated that six types of venom peptide/protein, that is to say “α-KTx, β-KTx, scorpine-like peptide, anionic peptide, La1-like peptide, and SPSV,” were likely recruited into the scorpion venom peptidome/protome before the lineage split between Euscorpiidae and Buthidae. After that, these recruited genes underwent individual or multiple gene duplication events, followed by gene mutations, which significantly enriched the molecular diversity of scorpion venom peptide/protein. Recently, He *et al.* performed a transcriptomic analysis of the venom glands from two scorpion species of the family Chaerilidae, *Chaerilus tricostatus* and *Chaerilus tryznai* [75]. Fourteen types of venom peptide/protein and 74 atypical venom molecules were discovered in two species. Surprisingly, the venom components of the family Chaerilidae were also found to have four toxin types (NaTx, β-KTx, Scamp (short cationic antimicrobial peptide) and BPP-like), which were previously considered to be specific to the family Buthidae. Moreover, the cytolytic peptide was the most abundant toxin type in the venom of the family Chaerilidae, sharing the same expression pattern in the family Euscorpiidae. Lastly, three toxin types of NaTx, β-KTx and BPP-like were recruited into the venom before the lineage split between Buthidae and non-Buthidae families by comparative transcriptome and molecular phylogenetic analyses. This paper made a clear fact that the family Chaerilidae has a unique venom arsenal that is different from either the family Buthidae or the other non-Buthidae families, which depicts the evolutionary trace of scorpion venom peptide/protein components from Buthidae to non-Buthidae. More recently, our lab decoded the draft genome sequence of the scorpion *M*. *martensii*, and found 116 genes encoding venom neurotoxins located in the *M*. *martensii* genome, including 61 NaTx (toxins for sodium channels), 46 KTx (toxins for potassium channels), five ClTx (toxins for chloride channels) and four CaTx (toxins for ryanodine receptors) genes [76] *et al*. In total, our group had constructed nine venom gland cDNA libraries covering eight scorpion species from China, and then performed their transcriptome analyses (Table 2). The work allows the characterization of a large family of venom molecules, belonging to either known or unknown scorpion venom peptide/proteins, which not only supplies solid clues for understanding the molecular diversity of scorpion venom peptide/protein, but also provides new sets of venom molecules with therapeutic potential. Forthermore, the work contributes to a majority of new toxin molecules from Chinese scorpion species, and shows insights into the evolution of the scorpion venom arsenal by comparison with venom data from other scorpion lineages, as well as helps us to understand the dynamic and functional evolution of scorpion species and their toxins (Figure 2).

## 3. Diverse Functions of Toxins from Chinese Scorpion Species

### 3.1. Na^+^ Channel Modulators

The NaTxs are a kind of toxin with 58–76 amino acid residues stabilized by four disulfide bonds, and they can modulate the inactivation and activation of the sodium channels by α-NaTxs and β-NaTxs, respectively [92]. The isolation and function of the different NaTxs from the scorpion *M*. *martensii* were reviewed early in 2002 [50]. Since then, there was some progress in characterizing the functions of NaTxs in China. As for NaTxs from the scorpion *M*. *martensii*, the toxin BmKIM was found to be toxic to both mammal and insects, and inhibits the sodium currents in rat doesal root ganglion neurons and ventricular myocytes. It also could protect against the cardiac arrhythmia in the rat model of the aconitine-induced arrhythmia [56]. The effect of toxin BmKI on the sodium channel currents in dorsal root ganglion neurons was investigated, and it was found that the inhibitory effect of BmKI on open-state inactivation of tetrodotoxin-sensitive sodium currents was stronger than that of tetrodotoxin-resistant sodium currents [93]. Toxin BmK alphaIV was cloned and expressed, and pharmacological experiments indicated that it could increase the peak amplitude and prolonged the inactivation phase of rNav1.2 channel currents [94]. Recently, toxin BmαTX14 was found to selectively inhibit the fast inactivation of mNa(v)1.4 (EC_50_ = 82.3 ± 15.7 nM) rather than that of rNa(v)1.2 (EC_50_ > 30 μM) [52]. Besides the scorpion *M*. *martensii*, the NaTxs from the *M*. *eupeus* were also studied. The two-electrode voltage-clamp recordings toxins revealed that toxin MeuNaTxα-1, -2, -4, and -5 slowed inactivation of DmNa(v)1 and were inactive on Nav1.8 at micromolar concentrations. Among other six Nav1.2~Nav1.7 channels, these toxins exhibited differential specificity [95]. In addition, few β-NaTxs from Chinse scorpions was characterized in past years, which will be an interesting suject in the future. In summary, the work further hightlights the functional diversity of the NaTxs from different scorpion species.

### 3.2. K^+^ Channel Blockers

The KTxs are usually blockers of different types of the potassium channels, and their molecular diversity was recently reviewed in the 2008 [96]. In past years, many KTxs from the scorpion *M*. *martensii* and *M*. *eupeus* were found to block different potassium channels [41,97,98]. However, remarkable advances were achieved in the scorpion toxin–potassium channel interactions at the structural level. Through combining the mutagenesis experiments and computational modeling, the different toxin–potassium channel-binding modes and their interaction details were characterized, such as BeKm-1 acting on the hERG channel [99], maurotoxin acting on Kv1.2 channel [100], charybdotoxin acting on BKCa channel in the presence and absence of β4 subunits [101,102], Kunitz-type toxins acting on Kv1.3 channel [103], BmP05 acting on SKCa3 channel [10]. Based on these toxin–potassium channel interactions, a novel control technique of toxin–potassium channel recognition was developed [104], and used to rationally design a selective ADWX-1 peptide for treating the Kv1.3 channel-mediated multiple sclerosis [103,105,106]. These progresses indicate the importance and prospects of the structural information on the scorpion toxin–potassium channel interactions.

### 3.3. Antimicrobial Peptides

The scorpion only has an innate immune system that enables it to resist microbial infections, which suggests that there are a variety of antibacterial peptides in the body of scorpion. In 1993, a scorpion defensin with a 4 kDa was purified and characterized from the scorpion *Leiurus quinquestriatus* [107]. Consequently, research interests mainly focused on the discovery of antibacterial peptides from scorpion venoms [108]. Since 2001, our group found a series of AMPs from the venoms of Chinese scorpion species, and identified their antibacterial functions and mechanisms [67,68,109,110,111].

A group of toxin precursors were characterized from the venom of the scorpion *M*. *martensii*, which were deduced to encode a novel venom peptide family: no disulfide bridge peptide (NDBP) with antimicrobial activity, such as BmKn1 and BmKn2. The synthetic BmKn2 was then confirmed to inhibit the growth of the standard bacteria [67]. Kn2-7 was derived from BmKn2 to show not only increased inhibitory activity against both standard bacteria and clinical antibiotic-resistant strains (such as methicillin-resistant *Staphylococcus aureus*: MRSA), but also reduced hemolytic activity. Moreover, Kn2-7 effectively protected the *S*. *aureus* mouse skin infection of mice. Kn2-7 exerted its bactericidal activity by binding to the lipoteichoic acid (LTA) in the *S*. *aureus* cell wall and the lipopolysaccharides (LPS) in the *E*. *coli* cell wall, respectively [111].

Mucroporin was the second representative of antimicrobial peptides from Chinese scorpion venoms [109]. Mucroporin was cloned and characterized from the venom of *L*. *mucronatus*. Mucroporin specifically inhibited the growth of Gram-positive bacteria. Subsequently, Mucroporin-M1 was designed from the molecular template of Mucroporin. Mucroporin-M1 not only had the higher antibacterial activity against both standard and clinic Gram-positive bacteria, but also displayed wider antibacterial spectra (effect on Gram-negative bacteria). 

Finally, Ctriporin was a new anti-methicillin-resistant *S*. *aureus* peptide from the venom of the scorpion *C*. *tricostatus* [112]. The MICs of Ctriporin against standard and clinical antibiotic-resistant Gram-positive bacterial strains were 5 to 20 μg/mL. Furthermore, external use of the peptide Ctriporin dramatically decreased the bacterial counts and cured skin infections in mice. Ctriporin was demonstrated to have antimicrobial activity via the bactericidal mechanism of rapid cell lysis. 

Combined, the other antimicrobial peptides from Chinese scorpion venoms were screened and identified by our group [113,114], and these works confirm that scorpion venom is a rich resource of AMPs, which opens a new window for discovering antimicrobial resources and agents, and sheds some light into developing antimicrobial drugs against multidrug-resistant pathogens that seriously threaten human health.

### 3.4. Protease Inhibitors

It is well known that scorpion venom contains a variety of peptides and proteins used as a molecular arsenal for predation and defense. Naturally, it is interesting how scorpions protect their venom peptides/proteins from degradation. Under the natural selection pressure, scorpions strive to construct more efficient toxic peptides/proteins by recruitment events so as to be evolutionarily successful [48,75]. Peptide protease inhibitors are also considered to be the earliest venom peptide/protein type recruited from the other body proteins, accompanied by the recruitment of peptide toxins.

Several protease inhibitors have been isolated from scorpion venom. First, a new Kunitz-type venom peptide gene precursor, SdPI, was cloned and characterized from a venom gland cDNA library of the scorpion *L*. *mucronatus* [115]. It codes for a signal peptide of 21 residues and a mature peptide of 59 residues. The recombinant SdPI peptide showed trypsin inhibitory activity with high potency Ki = 1.6 × 10^−7^ M and thermostability. SdPI is the first functionally characterized Kunitz-type trypsin inhibitor from scorpion venom, and it may represent a new class of Kunitz-type venom peptides. Second, one Ascaris-type protease inhibitor, SjAPI, was discovered from the venom of the scorpion *S*. *jendeki* [116]. SjAPI contains 64 residues with a classical Ascaris-type cysteine framework reticulated by five disulfide bridges, but is different from all known protease inhibitors from venomous animals. SjAPI has α-chymotrypsin- and elastase-inhibiting properties. SjAPI is the first functionally characterized animal toxin peptide with an Ascaris-type fold. Third, a Kunitz-type protease inhibitor (BmKPI) was characterized from the venom gland of the scorpion *M*. *martensii* [117]. BmKPI showed strong and wide inhibitory activity toward trypsin (Ki 1.8 × 10^−6^ M), chymotrypsin (Ki 3.2 × 10^−8^ M), and elastase (Ki 1.6 × 10^−7^ M). Cysteine mutagenesis indicated that the disulfide bridge Cys53–Cys61 has little effect on its inhibitory activity against elastase. Therefore, BmKPI is a new multifunctional serine protease inhibitor and is also the first functionally characterized Kunitz-type elastase inhibitor from scorpion venoms.

Proteases catalyze the breakdown of proteins, which is a normal process necessary to maintain cellular homeostasis. Both proteases and protease inhibitors are employed for medicinal and pharmaceutical purposes [118]. For example, trypsin is involved in many inflammatory reactions in the human body, such as pancreatitis and cardiovascular or nervous systems diseases [119,120]. Thus, trypsin inhibitors, such as ulinastatin and aprotinin, are used as an anti-inflammatory therapy in clinic. There is no doubt that this work suggests the fact that scorpion venom is a rich source of protease peptide inhibitors with a great deal of potential for therapeutic drugs.

## 4. Conclusions

The scorpion is one of the important arachnids in the phylum Arthropoda. It has unique features, such as poisonous venom, fluorescence and so on, which increasingly attract the attention and interest of scientists around the world. Recently, some work has expanded the understanding of the biological functions of scorpion toxins, such as the discovery of enzyme inhibitors in scorpion venoms. Using scorpion species and their toxins from China as an example, we try to build the bridge between scorpion species and their toxins, in order to help us understand the molecular and functional diversity of the scorpion venom arsenal, the dynamic and functional evolution of scorpion toxins, and the relationship of scorpion species and their toxins.
